# Isolation of cytoplasmic NADPH-dependent phenol hydroxylase and catechol-1,2-dioxygenase from *Candida tropicalis* yeast

**DOI:** 10.2478/v10102-010-0046-7

**Published:** 2010-11

**Authors:** Lenka Vilímková, Jan Páca, Veronika Kremláčková, Jan Páca, Marie Stiborová

**Affiliations:** 1 Department of Biochemistry, Faculty of Science, Charles University, Prague, Czech Republic; 2 Department of Fermentation Chemistry and Bioengineering, Institute of Chemical Technology, Prague, Czech Republic

**Keywords:** environmental pollutants, phenol, biodegradation, yeast, *Candida tropicalis*, NADPH-dependent phenol hydroxylase, catechol-1,2-dioxygenase

## Abstract

The efficiencies of NADPH-dependent phenol hydroxylase (EC 1.14.13.7) and catechol 1,2-dioxygenase (EC.1.13.11.1) in biodegradation of phenol in the cytosolic fraction isolated from yeast *Candida tropicalis* were investigated. Enzymatic activities of both NADPH-dependent phenol hydroxylase and catechol 1,2-dioxygenase were detected in the cytosolic fraction of *C. tropicalis* grown on medium containing phenol. Using the procedure consisting of chromatography on DEAE-Sepharose, fractionation by polyethylene glycol 6000 and gel permeation chromatography on Sepharose 4B the enzyme responsible for phenol hydroxylation in cytosol, NADPH-dependent phenol hydroxylase, was isolated from the cytosolic fraction of *C. tropicalis* close to homogeneity. However, fractionation with polyethylene glycol 6000 lead to a decrease in catechol 1,2-dioxygenase activity. Therefore, another procedure was tested to purify this enzyme. Gel permeation chromatography of proteins of the eluate obtained by chromatography on a DEAE-Sepharose column was utilized to separate phenol hydroxylase and catechol 1,2-dioxygenase. Among gel permeation chromatography on columns of Sephadex G-100, Sephacryl S-300 and Sepharose 4B tested for their efficiencies to isolate phenol hydroxylase and catechol 1,2-dioxygenase, that on Sephacryl S-300 was found to be suitable for such a procedure. Nevertheless, even this chromatographic method did not lead to obtain catechol 1,2-dioxygenase in sufficient amounts and purity for its further characterization. The data demonstrate the progress in resolving the enzymes responsible for the first two steps of phenol degradation by the *C. tropicalis* strain.

## Introduction

**Figure 1:**
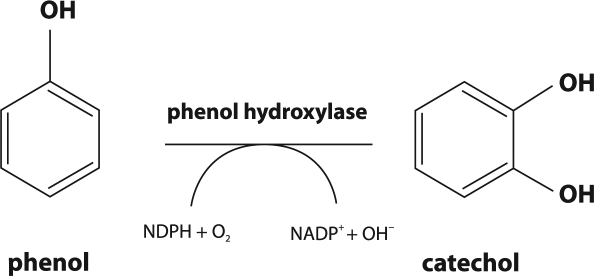
Phenol oxidation to catechol.

**Figure 2:**
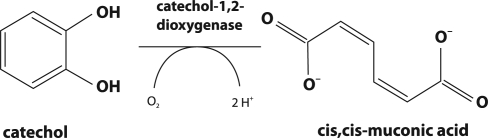
Catechol intra-diol cleavage to cis,cis- muconic acid.

Phenol and its derivatives are found in wide variety of wastewaters including those from the oil refining, petrochemical, coke and coal gasification industries. Removal of phenol from such wastewaters can be achieved through aerobic biodegradation in well-run activated sludge plants. *Pseudomonas* is a bacterial genus commonly found in such plants and *Pseudomonas putida* is a species capable of using phenol as a major source (Bayly and Wigmore, 1973; Yang and Humphrey, 1975). In addition, several other mesophilic bacteria are able to degrade phenol, including *Alcaligenes spp.* and *Spreptomyces setonii* and also the thermophile, *Bacillus stearothermophilus* (Gurujeyalakshmi and Oriel, 1989). Although bacteria are most likely to be responsible for aerobic breakdown of phenol in activated sludge, fungi including *Trichosporon cutaneum, Candida albicans* TL3 and *Candida tropicalis* are also capable of utilizing phenol as the major carbon source (Krug *et al*., 1985; Krug and Straube, 1986; Chang *et al*., 1998; Bastos *et al*., 2000; Komárková and Páca, 2000; Páca *et al*., 2002; Komárková *et al*., 2003; Stiborová et *al*., 2003; Ahuatzi-Chacon *et al*., 2004; Tsai *et al*., 2005). The aerobic degradation pathways in bacteria and yeast involve the occurrence of vicinal diols as substrates of ring-cleaving enzymes. Thus, the first step of phenol degradation is a hydroxylation of phenol to catechol (Figure 1). Catechol can undergo fission either by an intra-diol or an extra-diol type of cleavage (*ortho*- or *meta*-fission). *Meta*-fission leads to 2-hydroxymuconic semialdehyde and further to formate, acetaldehyde, and pyruvate. Such a catechol cleavage was not found in yeast. *Ortho*-fission, found in yeast such as *T. cutaneum, C. albicans* TL3 and *C. tropicalis*, gives rise to *cis,cis*-muconic acid (Figure 2), which is converted in further enzymatic steps *via* 3-oxoadipate to succinate and acetyl-CoA. These products enter the central metabolism of the cell (Krug *et al*., 1985; Krug and Straube, 1986; Bastos *et al*., 2000; Komárková and Páca, 2000; Páca *et al*., 2002; Komárková *et al*., 2003; Ahuatzi-Chacon *et al*., 2004; Tsai *et al*., 2005). Although examples are known in which the yeast *C. tropicalis* utilizes phenol for growth or metabolism (Krug *et al*., 1985; Krug and Straube, 1986; Stephenson, 1990; Chang *et al*., 1998; Bastos *et al*., 2000; Komárková and Páca, 2000; Páca *et al*., 2002; Komárková *et al*., 2003; Ahuatzi-Chacon *et al*., 2004) much less information on the nature of the phenol-oxidizing enzymes in this microorganism are known.

The enzymes responsible for the first step of degradation (the formation of catechol: Figure 1) in *C. tropicalis* yeast are: (i) cytochrome P450 (EC 1.14.15.1), the enzyme of the mixed function monooxygenase system localized in the membrane of endoplasmic reticulum (Stiborová *et al*., 2003; 2004) and (ii) cytoplasmic NADPH-dependent phenol hydroxylase (EC 1.14.13.7) (Krug *et al*., 1085; Krug and Straube, 1986; Xu *et al*., 2000; Stiborová *et al*., 2004; Páca *et al*., 2007). Indeed, recently, we have found that microsomal cytochrome P450 and cytosolic NADPH-dependent phenol hydroxylase are expressed in *C. tropicalis* grown on phenol and are capable of hydroxylation of phenol to form catechol (Stiborová *et al*., 2003; Páca *et al*., 2007). Cytosolic NADPH-dependent phenol hydroxylase seems to be the predominant enzyme responsible for the first step of phenol biodegradation in the *C. tropicalis* yeast; its activity is more than two orders of magnitude higher than that found in the microsomal fraction of this microorganism (Stiborová *et al*., 2003; Páca *et al*., 2007). During the second step of phenol degradation in *Candida* yeast, intra-diol cleavage of catechol to *cis,cis*- muconic acid (Figure 2) occurs (Bastos *et al*., 2000; Páca *et al*., 2002; Ahuatzi-Chacon *et al*., 2004; Tsai *et al*., 2005; 2007), being catalyzed by cytosolic catechol 1,2-dioxygenase (EC.1.13.11.1), the enzyme found in several microorganisms, including yeast (Nakai et al., 1990; Eck and Bettler, 1991; Briganti *et al*., 1997; Shen *et al*., 2004; Tsai and Li, 2007). NADPH-dependent phenol hydroxylase has already been purified from the cytosolic fraction of *C. tropicalis*.and partially characterized (Páca *et al*., 2007). However, information on catechol 1,2-dioxygenase of this microorganism are scarce. Even though the activity of this enzyme was detected in *C. tropicalis* cytosol (Ahuatzi-Chacon *et al*., 2004), its isolation from this microorganism has not been described as yet.

The aim of the present study was to develop the procedure to isolate both phenol hydroxylase and catechol 1,2-dioxygenase from the cytosolic fraction of *C. tropicalis*.

## Materials and methods

### Chemicals

Chemicals were obtained from the following sources: NADPH, catechol and bicinchoninic acid (2,2′-biquinoline-4,4′-dicarboxylic acid) from Sigma Chemical Co., (St. Louis, MO), DEAE-Sepharose, Sephacryl S-300, Sephadex G-100 and Sepharose 4B from Pharmacia (Uppsala). Other chemicals were supplied by Lachema (Brno). All chemicals were of reagent grade purity or better.

### Microorganisms, cultivation methods and preparation of microsomal and cytosolic fractions

The yeast *C. tropicalis* was isolated from soil contaminated with aromatic hydrocarbons and identified using the culture collection and Research Center (Brno, Czech Republic) (Komárková and Páca, 2000). The yeast culture was maintained on slope agar with mineral salts and glucose as a carbon and energy source at 4°C. The growth medium was BSM medium [4.3 g/l K_2_HPO_4_, 3.4 g/l KH_2_PO_4_, 2 g/l (NH_4_)_2_SO_4_, 0.34 g/l MgCl_2_.6 H_2_O] containing 350 g/l phenol as a sole carbon and energy source (phenol medium). Cell cultivations were carried out in shaking flasks using fed batch process with the growth medium containing phenol (see above) at 30 °C and pH 5.2 as described previously (Martius *et al*., 1996; Páca and Martius, 1996; Stiborová *et al*., 2003; Páca *et al*., 2007).

After separation, the cells were washed three times with distilled water and disintegrated using mechanical disruption of the cells in the presence of liquid nitrogen to obtain the cell-free homogenate. The isolation of the microsomal and cytosolic fractions from the *C. tropicalis* cell-free homogenate was carried out by differential centrifugation (Stiborová *et al*., 2003; Páca *et al*., 2007) by the procedure used for isolation of such subcellular fractions from rat liver (Stiborová et al., 1995, 2001a; b).

### Purification of NADPH-dependent phenol hydroxylase and catechol 1,2-dioxygenase from *C. tropicalis* cytosol

All operations were carried out at 4°C. For the first, the procedure which was used in our former study to isolate NADPH-dependent phenol hydroxylase was used (Páca *et al*., 2007). Briefly, cytosolic fraction of *C. tropicalis* (330 ml) was applied to a DEAE-Sepharose column (2.6×22 cm) equilibrated with 50 mM sodium phosphate buffer pH 7.6. The NADPH-dependent phenol hydroxylase was eluted using a linear gradient of 0–0.5 M NaCl in the same buffer. Phenol hydroxylase eluted at 0.1–0.15 M. Fractions containing phenol hydroxylase activities were pooled and precipitated by PEG 6000 (16% saturation). The precipitate was dissolved in 50 mM sodium phosphate buffer pH 7.6 (2 ml) and applied to a Sepharose 4B column (1.6×60 cm), previously equilibrated with the same buffer. The enzyme was eluted using 50 mM sodium phosphate buffer pH 7.6 and the fractions containing the phenol hydroxylase activity were pooled, frozen and stored at −20°C until used.

However, using precipitation of proteins with PEG 6000 (16% saturation), no catechol 1,2-dioxygenase activity was detectable. Therefore, proteins of eluate obtained by DEAE-Sepharose chromatography were lyophilized and enzymes additionally purified by gel permeation chromatography on columns (0.5×50 cm, bed volume 20 ml) of Sephadex G-100, Sephacryl S-300 and Sepharose 4B, equilibrated with 50 mM sodium phosphate buffer pH 7.6. Lyophilized proteins (2 mg), re-suspended in 200 µl of 50 mM sodium phosphate buffer (pH 7.6), were applied on a column and eluted with the same buffer.

The phenol hydroxylase and catechol 1,2-dioxygenase activities were followed by formation and consumption of catechol, respectively, measured with HPLC, using a column of Nucleosil 100-5 C18 (4×250 mm) as described previously (Stiborová *et al*., 2003; Páca *et al*., 2007). The major product formed by phenol hydroxylation was identified by comparison of its retention time with an authentic standard of catechol, having the retention time of 7.9 min and by mass and UV/vis absorbance spectroscopy. Mass spectra were recorded on a FINNIGAN MAT INCOS 50 (electron impact, 70 eV, low resolution, direct inlet). UV/vis spectra were recorded on a Hewlett-Packard 8453 diode array spectrophotometer (Stiborová *et al*., 2003; Páca *et al*., 2007).

## Results

**Figure 3:**
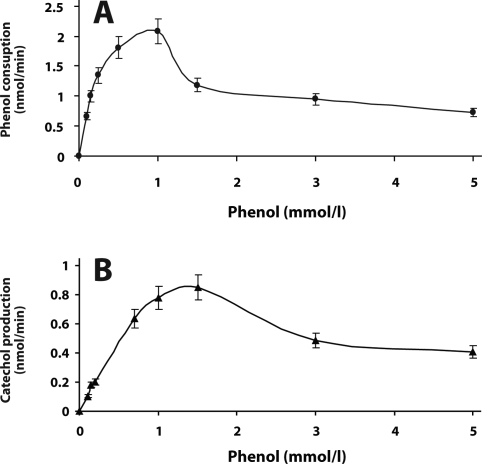
Substrate affinities of cytosolic enzymes of *C. tropicalis* towards phenol measured by disappearance of phenol (**A**) and formation of catechol (**B**).

The cytosolic fraction of *C. tropicalis* is able to oxidize phenol; a time-dependent decrease in phenol followed by an increase in formation of an oxidation metabolite, catechol, was found (data not shown). Non-Michaelian saturation curves were seen when the initial velocity of phenol oxidation catalyzed by the crude cytosolic fraction was plotted as a function of phenol concentrations (Figure 3A,B). The observed decrease in amounts of catechol generated in incubations containing the crude cytosolic fraction (Figure 3B), might be caused by its consumption with catechol 1,2-dioxygenase, whose activity was found by Ahuatzi-Chacon and collaborators (2004) in *C. tropicalis* cytosol. Therefore, these findings suggest the presence not only of NADPH-dependent phenol hydroxylase, but also catechol 1,2-dioxygenase in this subcellular fraction.

**Figure 4:**
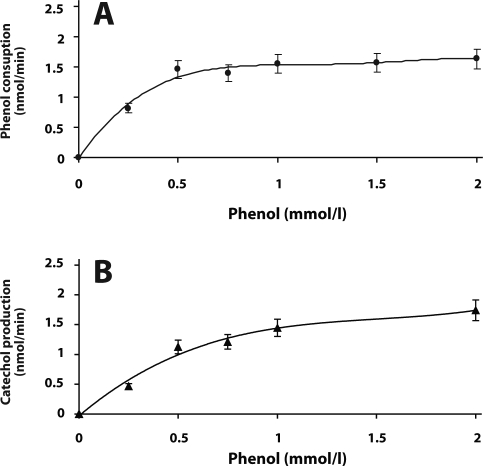
Substrate affinities of purified NADPH-dependent phenol hydroxylase of *C. tropicalis* towards phenol measured by disappearance of phenol (**A**) and formation of catechol (**B**).

Using the procedure described in our previous work (Páca *et al*., 2007), consisting of the chromatography on a DEAE-Sepharose column, fractionation with PEG 6000 and a gel filtration on Sepharose 4B (Table 1A), NADPH-dependent phenol hydroxylase was purified from the cytosolic fraction of *C. tropicalis* close to homogeneity. Figure 4 shows the kinetics of phenol oxidation by the purified enzyme. Phenol consumption and formation of catechol was measured in the reaction medium, which contained isolated NADPH-dependent phenol hydroxylase, NADPH and various concentrations of phenol. The reaction measured by both phenol consumption and formation of catechol followed the Michaelis-Menten kinetics (Figure 4). The Michaelian kinetics measured by formation of catechol confirms that the preparation of purified phenol hydroxylase is free of catechol 1,2-dioxygenase utilizing catechol as substrate. The values of a maximal velocity (V_max_) and an apparent Michaelis constant (K_m_) for oxidation of phenol are 54.3 nmol/min per mg of protein and 0.45 mmol/l, respectively. The values of V_max_ and an apparent K_m_ for oxidation of phenol determined from the formation of catechol are 54.4 nmol/min per mg of protein and 0.45 mmol/l, respectively.

Table 1Purification of NADPH-dependent phenol hydroxylase and catechol 1,2-dioxygenase from *C. tropicalis*.

**A:** 

Fraction	Volume	Proteins	Specific activity	
			
			phenol hydroxylase	catechol 1,2-dioxygenase
	(ml)	(mg/ml)	(nmol phenol/min/mg)	(nmol catechol/min/mg)
Cytosol	330.0	2.16	22.1	not measured
Eluate, DEAE-Sepharose	70.0	1.73	28.4	23.1
PEG 6000, ppt^a^ 0–16%	5.2	4.14	32.5	not detectable
Eluate, Sepharose 4B	13.8	1.15	41.5	not detectable

a ppt – precipitate

**B:** 

**Fraction**	**Specific activity**	
	
	phenol hydroxylase	catechol 1,2-dioxygenase
	(nmol phenol/min/mg)	(nmol catechol/min/mg)
Cytosol	22.1	not measured
Eluate, DEAE-Sepharose	28.4	23.1
Lyophilisate	not measured	not measured
Eluate, Sephadex G-100	14.8	54.8
Eluate, Sephacryl S-300	45.9	100.0
Eluate, Sepharose 4B	30.0	110.8

Experimental conditions are described in the *Material and methods* section.

**Figure 5:**
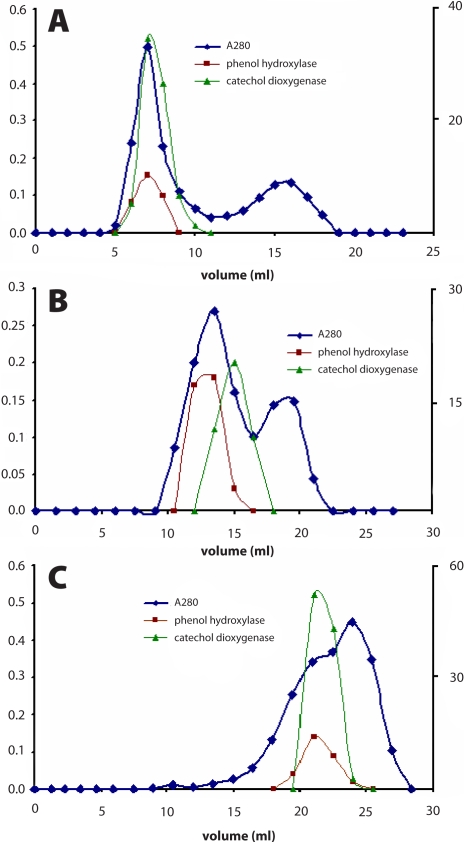
Chromatogram showing elution profile of phenol hydroxylase and catechol 1,2-dioxygenase on columns of Sephadex G-100 (**A**), Sephacryl S-300 (**B**) and Sepharose 4B (**C**)

While NADPH-dependent phenol hydroxylase and catechol 1,2-dioxygenase activities were detectable in the eluate obtained by the chromatography on a DEAE-Sepharose column, no activity of catechol 1,2-dioxygenase was detectable if proteins were precipitated with PEG 6000 (Table 1A). Therefore, another procedure was tested to obtain the enzymatically active catechol 1,2-dioxygenase. The proteins of pooled fractions containing phenol hydroxylase and catechol 1,2-dioxygenase activities, obtained by chromatography on DEAE-Sepharose, were lyophilized and used for additional enzyme purification. Gel permeation chromatography on columns of Sephadex G-100, Sephacryl S-300 and Sepharose 4B was utilized to separate phenol hydroxylase and catechol 1,2-dioxygenase (Figure 5). Using these procedures, besides protein fractions containing phenol hydroxylase and catechol 1,2-dioxygenase, the fraction of proteins having a lower molecular mass than both enzymes was eluted as a distinguish peak (Figure 5). Such a separation of ballast proteins led to an increase in specific activities of both enzymes (Table 1B). However, different efficiencies to separate phenol hydroxylase and catechol 1,2-dioxygenase were found for individual chromatography. The separation of both enzymes was reached only using chromatography on a column of Sephacryl S-300, whereas chromatography on Sephadex G-100 and Sepharose 4B was ineffective under the conditions used in the experiments.

## Discussion

Several fungi including *C. tropicalis* are capable of utilizing phenol as the sole carbon and energy source. These organisms might therefore be useful for biotechnological applications such as decontamination of phenol in wastewaters. The hydroxylation of phenol to catechol, and its additional intra-diol cleavage are the initial and rate-determining steps in the phenol degradation pathways in *Candida* yeast. The question which of the yeast enzymes are responsible for these two steps of phenol degradation in *C. tropicalis* yeast has not been fully answered yet.

Here, we demonstrate that NADPH-dependent phenol hydroxylase capable of oxidation of phenol to catechol and catechol 1,2-dioxygenase are present in cytosol of *C. tropicalis* yeast. Non-Michaelian saturation curves were seen when the initial velocity of phenol oxidation catalyzed by the crude cytosolic fraction was plotted as a function of phenol concentrations. Here, we show that the observed decrease in amounts of catechol generated in incubations containing the crude cytosolic fraction, is caused by its consumption with catechol 1,2-dioxygenase.

NADPH-dependent phenol hydroxylase was purified from the cytosolic fraction of *C. tropicalis* by the procedure consisting of chromatography on DEAE-Sepharose, fractionation by polyethylene glycol 6000 and gel filtration on Sepharose 4B and partially characterized. The K_m_ value of NADPH-dependent phenol hydroxylase of *C. tropicalis* for phenol is one order of magnitude higher than the value found for the enzyme from yeast *Trichosporon cutaneum* (Neujahr and Gaal, 1973). Although catechol 1,2-dioxygenase has recently been successfully isolated from *C. albicans* TL3 (Tsai and Li, 2007), and its activity was detected in *C. tropicalis* (Ahuatzi-Chacon *et al*., 2004), the enzyme has not been isolated from *C. tropicalis* as yet. Here, we tried to develop a procedure capable of separating catechol 1,2-dioxygenase from phenol hydroxylase, partially purified by chromatography on DEAE-Sepharose. The partial separation of both enzymes was reached using gel permeation chromatography on a column of Sephacryl S-300. Nevertheless, even this chromatographic method did not provide catechol 1,2-dioxygenase in sufficient amounts and purity for its further characterization. Therefore, additional procedures for its isolation are now tested in our laboratory.

In conclusion, the results presented in this paper demonstrate the ability of cytosolic NADPH-dependent phenol hydroxylase and catechol 1,2-dioxygenase of *C. tropicalis* to metabolize phenol, which is a contaminant of a wide variety of wastewaters. Here we assume that organisms rich in such enzymes might be able to degrade phenol and might be utilized in bioremediation technologies. The data shown in the paper are the first report showing isolation of both NADPH-dependent phenol hydroxylase and catechol 12,-dioxygenase from *C. tropicalis* and demonstrate the progress in resolving the enzymes responsible for the first steps of phenol degradation by the *C. tropicalis* strain.
